# Mortality by Admission Diagnosis in Children 1–60 Months of Age Admitted to a Tertiary Care Government Hospital in Malawi

**DOI:** 10.4269/ajtmh.22-0439

**Published:** 2023-06-20

**Authors:** Jason H. Choi, Thomas E. Tanner, Michelle D. Eckerle, Jane S. Chen, Emily J. Ciccone, Griffin J. Bell, Flexon F. Ngulinga, Elizabeth Nkosi, Rachel S. Bensman, Heather L. Crouse, Jeff A. Robison, Msandeni Chiume, Elizabeth Fitzgerald, on behalf of the Pediatric Alliance for Child Health Improvement in Malawi at Kamuzu Central Hospital and Environs (PACHIMAKE) Consortium

**Affiliations:** ^1^ Baylor International Pediatrics AIDS Initiative, Baylor College of Medicine, Houston, Texas;; ^2^ Division of Emergency Medicine, Cincinnati Children’s Hospital Medical Center, Cincinnati, Ohio;; ^3^ Section of Emergency Medicine, Department of Pediatrics, Baylor College of Medicine, Houston, Texas;; ^4^ Department of Pediatrics, University of Cincinnati College of Medicine, Cincinnati, Ohio;; ^5^ Institute for Global Health and Infectious Diseases, University of North Carolina, Chapel Hill, North Carolina;; ^6^ Division of Infectious Diseases, Department of Medicine, University of North Carolina School of Medicine, Chapel Hill, North Carolina;; ^7^ Department of Epidemiology, UNC Gillings School of Global Public Health, Chapel Hill, North Carolina;; ^8^ Department of Medicine, Kamuzu Central Hospital, Lilongwe, Malawi;; ^9^ Department of Pediatrics, Kamuzu Central Hospital, Lilongwe, Malawi;; ^10^ Division of Pediatric Emergency Medicine, Department of Pediatrics, University of Utah School of Medicine, Salt Lake City, Utah;; ^11^ Division of Emergency Medicine, Department of Pediatrics, University of North Carolina School of Medicine, Chapel Hill, North Carolina

## Abstract

Diagnosis-specific mortality is a measure of pediatric healthcare quality that has been incompletely studied in sub-Saharan African hospitals. Identifying the mortality rates of multiple conditions at the same hospital may allow leaders to better target areas for intervention. In this secondary analysis of routinely collected data, we investigated hospital mortality by admission diagnosis in children aged 1–60 months admitted to a tertiary care government referral hospital in Malawi between October 2017 and June 2020. The mortality rate by diagnosis was calculated as the number of deaths among children admitted with a diagnosis divided by the number of children admitted with the same diagnosis. There were 24,452 admitted children eligible for analysis. Discharge disposition was recorded in 94.2% of patients, and 4.0% (*N* = 977) died in the hospital. The most frequent diagnoses among admissions and deaths were pneumonia/bronchiolitis, malaria, and sepsis. The highest mortality rates by diagnosis were found in surgical conditions (16.1%; 95% CI: 12.0–20.3), malnutrition (15.8%; 95% CI: 13.6–18.0), and congenital heart disease (14.5%; 95% CI: 9.9–19.2). Diagnoses with the highest mortality rates were alike in their need for significant human and material resources for medical care. Improving mortality in this population will require sustained capacity building in conjunction with targeted quality improvement initiatives against both common and deadly diseases.

## INTRODUCTION

During the Millennium Development Goal era (1990–2015), the mortality rate of children younger than 5 years of age declined by more than half in sub-Saharan Africa.^1^ Much of this progress is attributed to the success of public health interventions, particularly those targeting malaria, malnutrition, and mother-to-child transmission of HIV.^2^^–^^4^ However, as the world moves into the Sustainable Development Goal era and aims to end all preventable under-5 deaths by 2030, public health strategies alone will not be sufficient.^5^^–^^7^ Child hospital mortality remains high, and challenges delivering high-quality hospital care may be responsible for over half of all deaths in low- and middle-income countries (LMICs).^8^^,^^9^ Assessing and improving the quality of pediatric hospital care will be crucial in reducing childhood mortality.

Assessments of pediatric healthcare quality in sub-Saharan Africa are limited. Baseline data on births and deaths are often incomplete due to unreliable civil registration and vital statistics systems.^10^ Many countries rely on cross-sectional surveys to evaluate healthcare quality measures, such as vaccination coverage.^11^ Hospital-level quality measures are incompletely characterized.^6^^,^^7^ Typically, hospital-level data describe aggregate mortality rates in children and diagnoses associated with the largest number of deaths.^8^^,^^12^^–^^15^ The cause-specific mortality rate, or case fatality rate, is often not reported because it is challenging to attribute a primary diagnosis in patients with multiple comorbidities in settings with limited diagnostic testing. Studies recruiting patients with a single disease can report these measures but are often unable to place the single disease mortality rate in context with the mortality rates of other diseases.^16^ An approach that approximates the mortality rates of multiple diseases at the same hospital may help clinicians and leaders evaluate the quality of healthcare and prioritize areas for improvement.^5^^–^^7^^,^^17^^,^^18^

In this study, we investigated mortality by diagnosis in children less than 5 years of age who were admitted to a tertiary care government referral hospital in Malawi. Most children in this cohort were admitted with multiple diagnoses, reflecting a degree of uncertainty that is not uncommon in sub-Saharan African healthcare settings. Without a primary diagnosis or cause of death, we do not report a cause-specific mortality rate or a case fatality rate. Instead, our study gives each diagnosis equal consideration and primarily reports mortality rates in children with a diagnosis regardless of their comorbidities.

## MATERIALS AND METHODS

### Study design and population.

This study was a secondary analysis of a prospective routine database of children admitted to the Kamuzu Central Hospital’s (KCH) Pediatric Department between October 1, 2017, and June 30, 2020.^19^ Before 2019, admissions of children aged 6–36 months were entered into the database. From January 2019, admissions of children aged 2 weeks to 60 months were entered. This analysis includes patients aged 1–60 months. Patients were excluded if they presented dead on arrival or had no documented admission diagnosis.

### Setting.

This study was conducted at KCH, a tertiary care government referral hospital located in Lilongwe, Malawi. It serves the central region of the country and has a catchment area of 7.5 million people.^20^ Most patients are referred to KCH from district health centers or regional hospitals, but they may also present directly for care. Therefore, KCH also provides services at the primary and secondary level.

During the study period, the pediatric department evaluated and admitted approximately 100,000 and 27,000 patients per year, respectively.^19^ Rotating medical interns and permanently employed pediatric-trained clinical officers evaluated and treated most patients. Between one and three consultant pediatricians provided daily clinical supervision. National and expatriate pediatricians with primary responsibilities outside of KCH provided limited additional supervision.

Diagnostic studies immediately available upon admission included point-of-care testing for malaria, glucose, and hemoglobin. Anthropometric measurements and malnutrition screening occurred at triage.^21^ Routine HIV testing was conducted after admission by a local partner.^22^ Other laboratory and radiologic investigations were inconsistently available.^8^ Inpatient therapies were provided free of charge and included oral rehydration fluids, intravenous fluids, pharmaceutical therapy, and ready-to-use therapeutic food. Critical care, including mechanical ventilation, was provided on a limited basis, and pediatric critical care specialists were generally unavailable.

### Admission diagnoses.

Admission diagnoses were made by clinicians and written into paper charts. Data clerks reviewed paper charts and entered all documented diagnoses into an electronic database. Clinicians could give patients an unlimited number of diagnoses and did not indicate a primary diagnosis in the chart. Therefore, all diagnoses were given equal consideration, and none were labeled as comorbidities or consequences of a primary diagnosis. We considered only diagnoses at admission, as opposed to at discharge or death. Future references to diagnoses in this manuscript refer to admission diagnoses unless otherwise indicated.

All diagnoses were clinical diagnoses and not verified with the use of other recorded measures in the database, such as hemoglobin levels, malaria rapid diagnostic testing results, or anthropometric measures. We felt that our approach provided a pragmatic description of clinician diagnostic patterns at admission. Other analyses including these recorded measures have been and will be published separately.^21^^,^^23^ Certain diagnoses were combined if considered clinically equivalent as follows. Pneumonia and bronchiolitis were combined as “pneumonia/bronchiolitis.” Dysentery and gastroenteritis were combined as “acute gastroenteritis (AGE).” “Malaria” included both uncomplicated and severe malaria. “Anemia” included anemia with and without malaria. Diagnoses that did not fit any category were labeled as “uncategorized diagnosis.” There were a total of 51 admission diagnoses including “uncategorized diagnosis.” Disposition was categorized as “discharged/home,” “absconded,” “death,” or “unknown.”

### Data analysis.

Demographic data, HIV status on discharge, referral status, and disposition were reported both in aggregate and stratified by age (1–5 months; 6–36 months; and 37–60 months, inclusive). These age groups were chosen because the database initially only contained data on children aged 6–36 months and was later expanded.

The number of diagnoses that patients had at admission and death were reported. The frequency of individual diagnoses at admission and death were also reported. The mortality rate by diagnosis was calculated as the number of deaths among children admitted with a diagnosis divided by the number of children admitted with the same diagnosis. Therefore, children with multiple diagnoses were counted more than once when analyzing mortality rate. A 95% binomial CI was calculated for each mortality rate using the normal approximation method. Only diagnoses with at least 0.5% prevalence were reported in the tables.

For the seven diagnoses of greatest prevalence, the mortality rate in patients with combinations of two diagnoses were calculated. For all diagnoses with at least 0.5% prevalence, we reported mortality rate by age group (1–5 months, 6–36 months, and 37–60 months). Finally, the mortality rate by referral status was reported with a 95% binomial CI calculated using the normal approximation method. Fisher’s exact test was used in pairwise comparisons between groups with the two-tailed Type I error set at 0.05. All analyses were performed using Stata 13.0 (Stata Corp, College Station, TX), R 4.2.2 Statistical Software (R Foundation for Statistical Computing, Vienna, Austria), or Excel 14.7.2 (Microsoft, Redmond, WA).

## RESULTS

**Figure 1.**
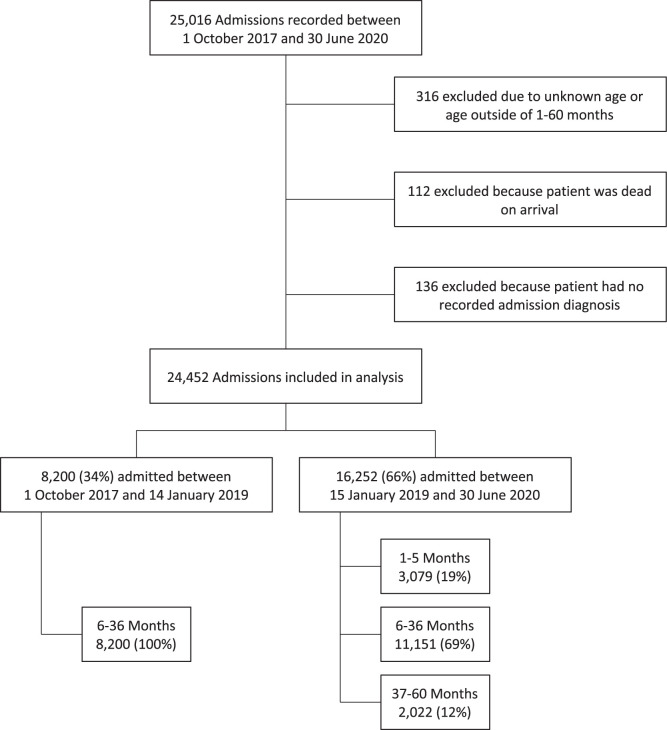
Distribution of patients in analysis.

Between October 1, 2017, and June 30, 2020, 25,016 admissions to the KCH Pediatric Department were recorded in the database. Patients were excluded if their age was outside of 1–60 months or unknown (*N* = 316), if they were dead on arrival (*N* = 112), or if they had no recorded admission diagnosis (*N* = 136) (Figure 1). Of the remaining 24,452 patients included in the analysis, 79.1% (*N* = 19,351) were in the 6- to 36-month age group (Table 1). The population was predominantly male (57.5%). The majority of children (80.5%, *N* = 19,669) had a known HIV status at discharge, with 1.7% (*N* = 409) confirmed as HIV infected. Most patients (76.1%, *N* = 18,619) initially presented to another facility before being referred to KCH. Disposition was recorded in 94.2% of patients (*N* = 23,043): 88.2% (*N* = 21,560) were discharged home, 2.1% (*N* = 506) absconded, and 4.0% (*N* = 977) died while in the hospital.

**Table 1:** Characteristics of the patient cohort stratified by age

Characteristic	Total	1–5 Months	6–36 Months	37–60 Months
Patients, *n* (%)	24,452 (100.0)	3,079 (12.6)	19,351 (79.1)	2,022 (8.3)
Sex, *n* (%)				
Female	10,360 (42.4)	1,262 (41.0)	8,209 (42.4)	889 (44.0)
Male	14,053 (57.5)	1,815 (58.9)	11,107 (57.4)	1,131 (55.9)
Unknown	39 (0.2)	2 (0.1)	35 (0.2)	2 (0.1)
Age in months, median (Q1, Q3)	16 (9, 26)	3 (1, 4)	17 (11, 25)	48 (42, 52)
HIV status at discharge, *n* (%)				
Infected	409 (1.7)	59 (1.9)	321 (1.7)	29 (1.4)
Uninfected, ongoing exposure	399 (1.6)	126 (4.1)	271 (1.4)	2 (0.1)
Uninfected, unexposed	18,861 (77.2)	2,350 (76.3)	14,917 (77.1)	1,594 (78.8)
Unknown	4,783 (19.6)	544 (17.7)	3,842 (19.9)	397 (19.6)
Referral status, *n* (%)				
Referred	18,619 (76.1)	2,453 (79.7)	14,618 (75.5)	1,548 (76.6)
Self-presented	5,575 (22.8)	603 (19.6)	4,529 (23.4)	443 (21.9)
Unknown	258 (1.1)	23 (0.7)	204 (1.1)	31 (1.5)
Disposition, *n* (%)				
Home	21,560 (88.2)	2,708 (88.0)	17,094 (88.3)	1,758 (86.9)
Death	977 (4.0)	150 (4.9)	737 (3.8)	90 (4.5)
Absconded	506 (2.1)	33 (1.1)	454 (2.3)	19 (0.9)
Unknown	1,409 (5.8)	188 (6.1)	1,066 (5.5)	155 (7.7)

There were 39,183 admission diagnoses assigned to the 24,452 admitted patients: 53.3% (*N* = 13,026) had one diagnosis, 35.4% (*N* = 8,664) had two diagnoses, and 11.3% (*N* = 2,762) had three or more diagnoses. There were 2,078 diagnoses assigned to the 977 patients who died: 28.7% (*N* = 280) had one diagnosis, 41.0% (*N* = 401) had two diagnoses, and 30.2% (*N* = 296) had three or more diagnoses.

The most frequent diagnoses among admissions and deaths were pneumonia/bronchiolitis, malaria, and sepsis (Tables 2 and 3). For diagnoses occurring in more than 0.5% of admissions, the highest mortality rates were found in surgical conditions (16.1%; 95% CI: 12.0–20.3), malnutrition (15.8%; 95% CI: 13.6–18.0), and congenital heart disease (14.5%; 95% CI: 9.9–19.2) (Table 4). When diagnoses of any prevalence were considered, the highest mortality rate were found in shock (57.1%; 95% CI: 36.0–78.3), respiratory failure (27.3%; 95% CI: 8.7–45.9), and hydrocephalus (23.7%; 95% CI: 15.0–32.2).

**Table 2:** Frequency of diagnoses among admissions

Rank	Diagnosis	Frequency, *n* (%)*
1	Pneumonia/bronchiolitis	10,680 (43.7)
2	Malaria	8,871 (36.3)
3	Sepsis	4,629 (18.9)
4	Anemia	3,375 (13.8)
5	Acute gastroenteritis	3,328 (13.6)
6	Dehydration	1,656 (6.8)
7	Malnutrition	1,054 (4.3)
8	Convulsions (without malaria)	954 (3.9)
9	Meningitis	694 (2.8)
10	Asthma	489 (2.0)
11	Uncategorized diagnosis	425 (1.7)
12	Surgical condition	304 (1.2)
13	Fever	268 (1.1)
14	Congenital heart disease	220 (0.9)
15	Dermatologic condition	204 (0.8)
16	Upper respiratory tract infection	172 (0.7)
17	Poisoning	132 (0.5)

* Number of admissions with diagnosis and percentage of admissions with diagnosis. Percentages do not add up to 100% because of multiple diagnoses per admission.

**Table 3:** Frequency of diagnoses among deaths

Rank	Diagnosis	Frequency, *n* (%)*
1	Malaria	390 (39.9)
2	Pneumonia/bronchiolitis	288 (29.5)
3	Sepsis	227 (23.2)
4	Anemia	222 (22.7)
5	Acute gastroenteritis	221 (22.6)
6	Dehydration	171 (17.5)
7	Malnutrition	167 (17.1)
8	Meningitis	62 (6.3)
9	Surgical condition	49 (5.0)
10	Uncategorized diagnosis	36 (3.7)
11	Congenital heart disease	32 (3.3)
12	Convulsions (without malaria)	25 (2.6)
13	Dermatologic condition	19 (1.9)
14	Upper respiratory tract infection	5 (0.5)
15	Fever	3 (0.3)
16	Asthma	3 (0.3)
17	Poisoning	0 (0.0)

* Number of deaths with diagnosis and percentage of deaths with diagnosis. Percentages do not add up to 100% because of multiple diagnoses per death.

**Table 4:** Mortality rate by diagnosis

Rank	Diagnosis	Mortality rate, %* (95% CI)
1	Surgical condition	16.1 (12.0–20.3)
2	Malnutrition	15.8 (13.6–18.0)
3	Congenital heart disease	14.5 (9.9–19.2)
4	Dehydration	10.3 (8.9–11.8)
5	Dermatologic condition	9.4 (5.4–13.4)
6	Meningitis	8.9 (6.8–11.1)
7	Uncategorized diagnosis	8.5 (5.8–11.1)
8	Acute gastroenteritis	6.6 (5.8–7.5)
9	Anemia	6.6 (5.7–7.4)
10	Sepsis	4.9 (4.3–5.5)
11	Malaria	4.4 (4.0–4.8)
12	Upper respiratory tract infection	2.9 (0.4–5.4)
13	Pneumonia/bronchiolitis	2.7 (2.4–3.0)
14	Convulsions (without malaria)	2.6 (1.6–3.6)
15	Fever	1.1 (0.0–2.4)
16	Asthma	0.6 (0.0–1.3)
17	Poisoning	0.0 (0.0–0.0)

* Percentages do not add up to 100% because of multiple diagnoses per admission.

The most frequent combinations of diagnoses were malaria and anemia (*N* = 2,965), AGE and dehydration (*N* = 1,551), and malaria and sepsis (*N* = 1,363). Children with pneumonia/bronchiolitis, malaria, sepsis, and AGE had lower mortality rates when admitted with these diagnoses alone than when admitted with multiple diagnoses (Table 5). Anemia, dehydration, and malnutrition were notable for particularly high mortality rates when in combination with other diagnoses. For example, the mortality rate of children admitted with AGE and no other diagnosis was 1.3% (95% CI: 0.2–2.4). However, the mortality rates of children admitted with AGE and anemia, AGE and dehydration, and AGE and malnutrition were 14.9% (95% CI: 8.5–21.2), 9.5% (95% CI: 8.1–11.0), and 22.5% (95% CI: 17.5–27.6), respectively.

**Table 5:** Mortality rates of combinations of diagnoses, % (95% CI)

Diagnosis	Co-diagnosis						
	No other diagnosis	Malaria	Sepsis	Anemia	AGE	Dehydration	Malnutrition
PNA/B	1.3 (1.0–1.5)	6.1 (4.6–7.5)	5.0 (3.5–6.6)	*14.7 (10.3–19.1*)	8.6 (6.2–11.0)	*18.6 (12.8–24.4)*	*16.8 (11.7–21.9)*
Malaria	2.1 (1.6–2.6)		4.8 (3.6–5.9)	6.0 (5.1–6.9)	9.8 (7.4–12.3)	*16.6 (11.6–21.6)*	*13.1 (9.0–17.2)*
Sepsis	2.4 (1.5–3.4)			*10.1 (7.0–13.2)*	6.6 (5.1–8.2)	*12.1 (8.9–15.3)*	*17.5 (12.5–22.5)*
Anemia	5.7 (0.8–10.5)				*14.9 (8.5–21.2)*	**28.9 (15.6–42.1)**	*10.5 (5.5–15.5)*
AGE	1.3 (0.2–2.4)					9.5 (8.1–11.0)	**22.5 (17.5–27.6)**
Dehydration	*12.5 (0.0–28.7)*						**29.2 (22.0–36.4)**
Malnutrition	*16.3 (10.5–22.2)*						

AGE = acute gastroenteritis; PNA/B = pneumonia/bronchiolitis. Normal type = 0.0–9.9%; italic type = 10.0–19.9%; bold type = ≥ 20.0%.

When stratified by age group, the overall mortality rate was higher in the 1- to 5-month group (4.9%; 95% CI: 4.1–5.6) compared with the 6- to 36-month group (3.8%; 95% CI: 3.5–4.1; *P* = 0.006) (Table 6). There were no other differences in overall mortality rate in pairwise comparisons by age group. The mortality rates for malaria, sepsis, and anemia were higher in the 1- to 5-month group compared with both the 6- to 36-month and 37- to 60-month age groups. The mortality rates of pneumonia/bronchiolitis and meningitis were higher in the 1- to 5-month group compared with the 6- to 36-month group. The mortality rate of AGE was higher in the 1- to 5-month group compared with the 37- to 60-month group, whereas the mortality rate of surgical conditions was higher in the 37- to 60-month group compared with the 1- to 5-month group. The mortality rate of dermatologic conditions was higher in the 37- to 60-month group compared with the 6- to 36-month group. All other pairwise comparisons of mortality rate by diagnosis between age groups were not statistically significant.

**Table 6:** Mortality rates of diagnoses by age

Diagnosis	Mortality rate, % (95% CI)			Age group comparisons,* P* value*		
	1–5 Months	6–36 Months	37–60 Months	1–5 vs. 6–36	1–5 vs. 37–60	6–36 vs. 37–60
All diagnoses	4.9 (4.1–5.6)	3.8 (3.5–4.1)	4.5 (3.6–5.4)	0.006	0.500	0.163
Pneumonia/bronchiolitis	3.5 (2.7–4.2)	2.5 (2.2–2.8)	2.2 (0.9–3.6)	0.009	0.194	0.876
Malaria	8.0 (4.4–11.7)	4.3 (3.9–4.8)	4.3 (3.1–5.4)	0.016	0.024	1.000
Sepsis	9.1 (6.4–11.8)	4.5 (3.8–5.1)	4.3 (2.3–6.3)	< 0.001	0.006	1.000
Anemia	14.2 (7.5–20.8)	6.5 (5.6–7.4)	5.3 (3.1–7.4)	0.005	0.005	0.390
Acute gastroenteritis	9.2 (5.6–12.7)	6.6 (5.7–7.5)	2.7 (0.0–5.6)	0.117	0.027	0.116
Dehydration	11.9 (6.4–17.3)	10.2 (8.7–11.8)	9.1 (0.6–17.6)	0.555	0.785	1.000
Malnutrition	22.6 (11.4–33.9)	15.6 (13.3–18.0)	14.1 (6.7–21.5)	0.178	0.249	0.875
Convulsions (without malaria)	2.9 (0.0–8.6)	2.6 (1.5–3.7)	2.5 (0.0–5.2)	0.605	1.000	1.000
Meningitis	18.6 (7.0–30.2)	8.2 (5.9–10.5)	8.8 (3.3–14.3)	0.045	0.155	0.845
Asthma	0.0 (0.0–0.0)	0.5 (0.0–1.3)	0.8 (0.0–2.5)	1.000	1.000	0.575
Uncategorized diagnosis	6.3 (3.2–9.3)	9.1 (7.4–10.8)	7.7 (4.4–11.0)	0.624	1.000	1.000
Surgical condition	12.2 (8.6–15.8)	17.8 (15.0–20.6)	29.0 (20.9–37.2)	0.285	0.047	0.147
Fever	0.0 (0.0–0.0)	1.3 (0.5–2.0)	0.0 (0.0–0.0)	1.000	1.000	1.000
Congenital heart disease	16.9 (12.5–21.3)	13.0 (10.0–15.9)	16.7 (7.9–25.5)	0.529	1.000	0.712
Dermatologic condition	5.9 (1.8–9.9)	7.5 (5.3–9.7)	25.0 (16.2–33.8)	1.000	0.055	0.018
Upper respiratory tract infection	10.0 (3.3–16.7)	2.2 (0.9–3.4)	0.0 (0.0–0.0)	0.122	0.496	1.000
Poisoning	0.0 (0.0–0.0)	0.0 (0.0–0.0)	0.0 (0.0–0.0)	1.000	1.000	1.000

* Fisher’s exact test.

In patients who were referred, self-presented, or had an unknown referral status, the mortality rates were 3.8% (95% CI: 3.7–3.9), 3.4% (95% CI: 3.2–3.6), and 29.1% (95% CI: 26.3–31.9), respectively. Patients with an unknown referral status accounted for 1.1% (258/24,452) of all admitted patients but 7.6% of all deaths (75/977). Pairwise comparison of the mortality rate between referred and self-presenting patients was not statistically significant (*P* = 0.136). Pairwise comparisons between patients with an unknown referral status and either referred or self-presenting patients were statistically significant (*P* < 0.001).

## DISCUSSION

In our study, we found that the mortality rate of hospitalized Malawian children varies widely depending on their admission diagnoses. The overall mortality rate and prevalence of diagnoses approximate the results of prior studies from Malawi.^8^^,^^12^^,^^13^^,^^24^ Children with surgical conditions, malnutrition, congenital heart disease, and dehydration had the highest mortality rates in our analysis. Children with the co-diagnoses of anemia, dehydration, and malnutrition also had higher mortality rates, which may indicate that these diagnoses are pathogenic consequences of other diagnoses and are markers of illness severity.

Our study differs from other studies of child hospital mortality in sub-Saharan Africa in its approach to diagnostic uncertainty. Most studies describing the mortality rates of hospitalized children report a single cause of death or final diagnosis.^13^^,^^25^^–^^36^ However, children in this population have comorbidities, such as HIV and severe acute malnutrition, that affect mortality. Furthermore, due to limited testing, imaging, and subspeciality consultation, many diagnoses can be challenging to confirm. Our study considers each diagnosis to have equal impact on mortality. We first report the mortality rate of children with a diagnosis regardless of co-diagnoses. We also then report the mortality rate of children admitted with combinations of diagnoses. Only one prior study has reported mortality rates in children with combinations of diagnoses.^14^ Our study also relies on clinical diagnoses. Single disease studies typically ensure the accuracy of the diagnosis by enrolling patients with strict inclusion and exclusion criteria meeting the case definition of a disease.^16^^,^^37^ However, they are costly to perform and do not depict the mortality burden of the entire hospitalized cohort. Our approach may provide a greater breadth of information, thereby helping leaders more quickly respond to the needs of the population.

Our study also differs from others because it reports mortality rates based on diagnoses at admission instead of at final disposition. Because treatment decisions in resource-limited settings are usually made at admission, our approach may provide clinicians a practical estimation of risk at the time of clinical decision-making. Furthermore, diagnoses at final disposition are not necessarily more accurate than admission diagnoses, and the concordance between the two remains unstudied in resource-limited settings.^38^^–^^43^

When examining the diagnoses with the highest mortality rates, they appear alike in their need for significant human and material resources for medical care. Malnutrition and dehydration both have standardized treatment plans that are effective in LMICs.^44^ However, in settings with inconsistent nursing coverage, the feeding or rehydration plan may be informally task shifted to guardians.^45^^–^^47^ Formal training of guardians and increased nursing coverage could improve adherence to these plans, thereby improving outcomes. Task-shifting of asthma education to lay educators, for example, has already been demonstrated in Malawi.^48^ For children with surgical conditions, both limited surgical capacity nationally and inconsistent clinical coverage of surgical patients in the hospital may contribute to their high mortality.^49^ In response, our department has considered increasing clinical coverage of the surgical wards with pediatric staff to help monitor these patients. Similarly, for patients with congenital heart disease, the inconsistent availability of subspecialists likely drives lethality. At our institution, only a few children have access to a pediatric cardiologist, and even fewer can be referred for definitive surgical care taking place outside of the country.^50^^,^^51^ Finally, when examining diagnoses of high mortality regardless of prevalence (i.e., shock, respiratory failure, and hydrocephalus), the need for consistent specialized physician and nursing care is again clear. Patients with these conditions typically require admission to intensive care units, which are lacking throughout sub-Saharan Africa.^52^

Late referral of patients may also drive the high mortality rates of some of these conditions. Although many children can obtain sufficient care at a local center, others require prompt recognition by the local clinician to refer the patient to a tertiary care facility. Unfortunately, delays in referral are frequent due to barriers in diagnostic testing, local expertise, and transportation.^53^^–^^56^ When these patients do arrive, they often present with advanced disease. Such patients would likely have high mortality rates even in resource-abundant settings. Our study did not find that referred patients and self-presenting patients had a difference in mortality. However, a disproportionate share of patients who died had an unknown referral status, which could affect our results. Therefore, further research on the impact of referral systems on child hospital mortality is necessary.

There are several limitations to our study. First, due to our methodology, our estimates of mortality rate by diagnosis may be less accurate compared with those reported by studies with strict case definitions. However, our study’s reported mortality rate of pneumonia/bronchiolitis is consistent with case fatality rates reported by cohort studies from Malawi.^16^^,^^57^^,^^58^ Unfortunately, there are few other cohort studies of hospitalized Malawian children with which to compare the accuracy of our other results. Second, our study is unable to identify deaths after discharge.^59^^–^^61^ Our reported mortality rates probably underestimate the true mortality burden of disease. Finally, 5.8% of patients in our study had an unknown disposition. Although this is a degree of data loss seen in other studies from sub-Saharan African hospitals, the possibility of missed deaths may cause our study to underestimate the true mortality rate.^15^

## CONCLUSION

Child hospital mortality in resource-limited settings is unacceptably high but is not insurmountable.^62^^,^^63^ By measuring the mortality rates of all prevalent conditions, hospitals can evaluate the quality of their care and track the impact of interventions. Throughout sub-Saharan Africa and within our own hospital, quality improvement initiatives, audits, post-graduate training programs, and clinical information systems are being established to reduce child hospital mortality.^12^^,^^14^^,^^64^^–^^67^ Accurate measurement of the impacts of these interventions will be essential in allocating the limited resources in these settings.^5^
